# Apoptosis-like cell death upon kinetoplastid induction by compounds isolated from the brown algae *Dictyota spiralis*

**DOI:** 10.1186/s13071-021-04693-7

**Published:** 2021-04-12

**Authors:** Olfa Chiboub, Ines Sifaoui, Manef Abderrabba, Mondher Mejri, José J. Fernández, Ana R. Díaz-Marrero, Jacob Lorenzo-Morales, José E. Piñero

**Affiliations:** 1 Instituto Universitario de Enfermedades Tropicales y Salud Pública de Canarias, Universidad de La Laguna (ULL), Avda. Astrofísico Fco. Sánchez S/N, 38203 La Laguna, Tenerife Spain; 2 Departamento de Obstetricia y Ginecología, Pediatría, Medicina Preventiva y Salud Pública, Toxicología, Medicina Legal y Forense y Parasitología, Universidad de La Laguna (ULL), La Laguna, Tenerife Spain; 3 Instituto Universitario de Bio-Orgánica Antonio González, Universidad de La Laguna (ULL), Avda. Astrofísico Fco. Sánchez 2, 38206 La Laguna, Tenerife Spain; 4 Laboratoire Matériaux-Molécules et Applications, University of Carthage, La Marsa, Carthage, Tunisia; 5 Red de Investigación Cooperativa en Enfermedades Tropicales (RICET), Madrid, Spain; 6 Departamento de Química Orgánica, Universidad de La Laguna (ULL), Avda. Astrofísico Fco. Sánchez S/N, 38203 La Laguna, Tenerife Spain

**Keywords:** *T. cruzi*, *L. amazonensis*, Diterpenes, Marine compounds, Antikinetoplastid, Apoptosis-like

## Abstract

**Background:**

The* in vitro* activity of the brown seaweed *Dictyota spiralis* against both *Leishmania amazonensis* and *Trypanosoma cruzi* was evaluated in a previous study. Processing by bio-guided fractionation resulted in the isolation of three active compounds, classified as diterpenes. In the present study, we performed several assays to detect clinical features associated to cell death in *L. amazonensis* and *T. cruzi *with the aim to elucidate the mechanism of action of these compounds on parasitic cells.

**Methods:**

The aims of the experiments were to detect and evaluate specific events involved in apoptosis-like cell death in the kinetoplastid, including DNA condensation, accumulation of reactive oxygen species and changes in ATP concentration, cell permeability and mitochondrial membrane potential, respectively, in treated cells.

**Results:**

The results demonstrated that the three isolated diterpenes could inhibit the tested parasites by inducing an apoptosis-like cell death.

**Conclusions:**

These results encourage further investigation on the isolated compounds as potential drug candidates against both *L. amazonensis* and *T. cruzi.*

**Graphic abstract:**

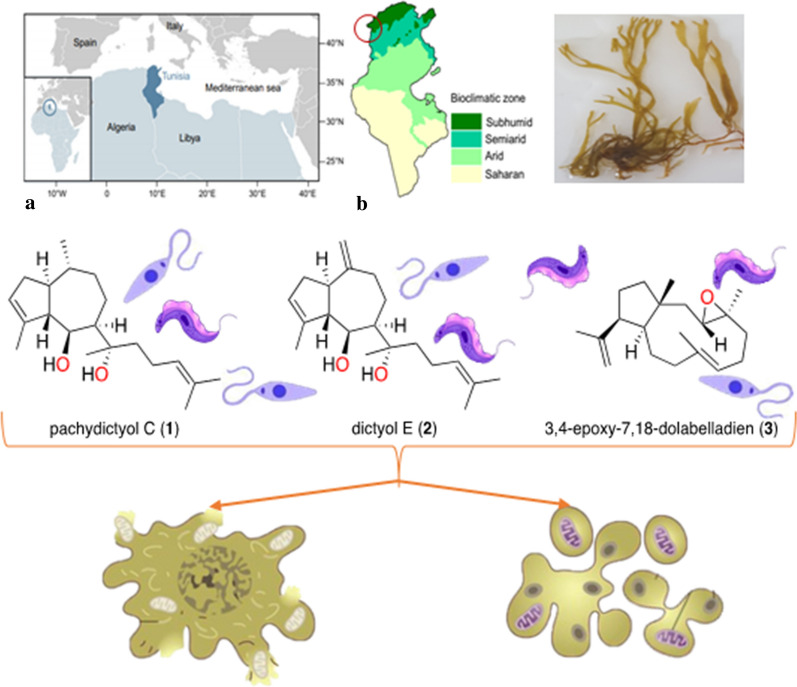

**Supplementary Information:**

The online version contains supplementary material available at 10.1186/s13071-021-04693-7.

## Background

Kinetoplastid parasites are responsible for a wide variety of infectious diseases, including *Leishmania* spp. and *Trypanosoma* spp., which are causative agents of tropical diseases classified as neglected by the World Health Organization. These diseases affect mainly countries with limited resources. The appearance of resistance to existing treatments as well as the side effects of these treatments motivate the search for new molecules as therapeutic alternatives [1, 2]. In this context, seaweeds could represent a good candidate as a source of new compounds. These marine organisms are well known to produce a wide variety of secondary metabolites and have been investigated in several studies and shown to exhibit various biological activities. Seaweeds have been source material for the production of a variety of major metabolites that can be classified in different groups according to their structure, such as polyphenols, polysaccharides, carotenoids, vitamins, lipids and terpenoid compounds, among others [3].

Terpenes are secondary metabolites with two or more five-carbon units of isopentenyl [4]. This class of molecules has been identified as being biologically active, with a proven efficiency as anticancer, antioxidant, anti-inflammatory and antimicrobial properties. They are also known to represent the major class of metabolites produced by marine algae [5].

Investigations on marine metabolites keep leading the discovery of new structures and new activities. Over 170 diterpenoids, included in 32 patterns of carbon backbones, have been found to exhibit biological activities, such as ichtyotoxicity, phytotoxicity, cytotoxicity, antiviral, antifungal, insecticidal or antifeeding properties [6–8].

The potential of these marine organisms to be a source of new biologically active molecules has inspired several investigations in drug discovery, including one on the screening of seaweed species collected from the Tunisian coast for antioxidant and antiprotozoal activities [9]. The results of this investigation allowed us to focus on the species *Dictyota spiralis* and its crude organic extract that exhibited promising results in terms of antikinetoplastid capacity. A further investigation employing bio-guided fractionation resulted in the isolation of specific compounds possessing this activity [10]. In this context, the research presented here focuses on the evaluation of the potential of these compounds as drug candidates. Therefore, the main objective was to identify the mechanism of action of these compounds on parasites at a molecular level and to characterize the cell death pathway they induce as the latter is a determinant parameter in drug selection as a prospective treatment. Cell death can be induced in two main forms: regulated or accidental. The first form is a modulated mechanism and the second form is instantaneous and catastrophic. Both pathways can be identified through the detection of macroscopic, morphological and molecular events [11].

The aim of the present work was to detect the various events induced by exposing the kinetoplasts of cells to three different metabolites isolated from the crude extract of *D. spiralis* and to determine the cell death pathway induced by these molecules.

## Methods

### Biological material and molecules identification

**Fig. 1:**
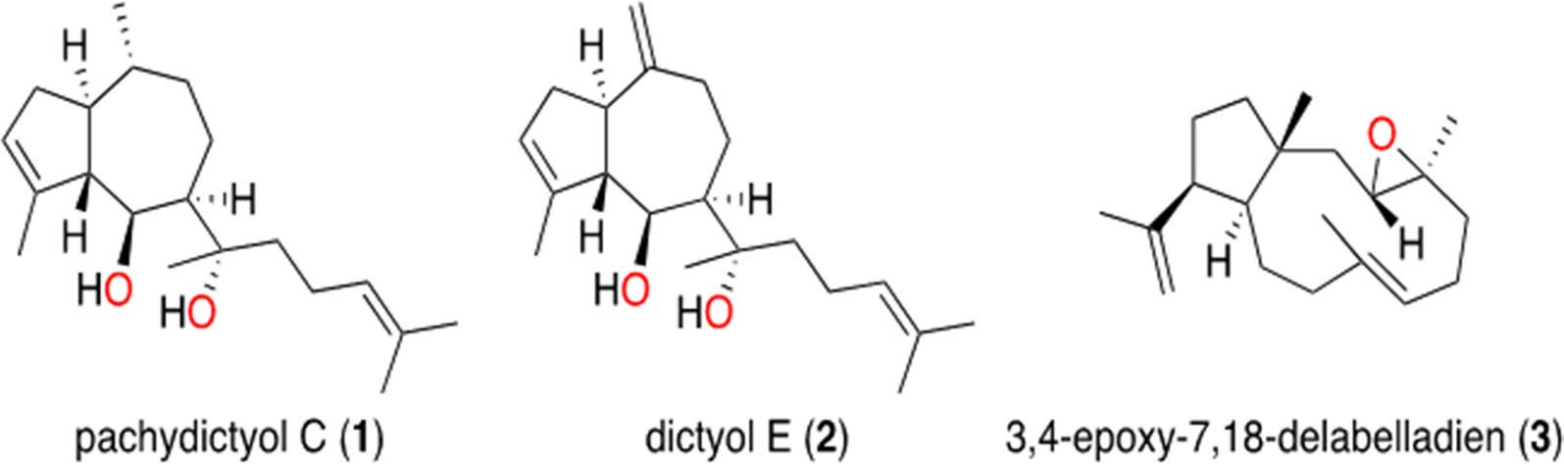
Chemical structure of three natural diterpenes isolated from *Dictyota spiralis* crude extract

The brown seaweed* Dictyota spiralis* was collected in April 2017, and specimen identification was performed by the National Institute for Marine Sciences and Technologies in Tunisia. A crude extract of the collected specimens was demonstrated to exhibit antikinetoplastid activity and was used to perform bio-guided fractionation to isolate the active compounds. Several methods of separation and identification were used, including column chromatography, high-performance liquid chromatography, nuclear magnetic resonance, mass spectrometry, among others (Additional file 1). Compound **1** was identified as pachydictyol C [12], and compounds **2** and **3** were characterized as dictyol E [12–14] and 3,4-epoxy-7,18-dolabelladiene [10], respectively. Stock solutions of these compounds were prepared in dimethyl sulfoxide (DMSO) (Merck KGaA, Darmstadt, Germany), protected from light, at − 20 °C. The structure as well as the chemical names of the three diterpenes obtained are shown in Fig. 1.

### Parasites strain, cytotoxicity and activity assays

Experiments including* in vitro* activity assays of isolated compounds **1**–**3** were performed against the promastigote and amastigote stages of *Leishmania amazonensis* (MHOM/BR/77/LTB0016) and the epimastigote stage of *Trypanosoma cruzi* (Y strain). Promastigotes of *L. amazonensis* were cultured in Schneider’s medium (Sigma-Aldrich, St. Louis, MO, USA). *Typanosoma cruzi* strain Y was cultured in liver Infusion tryptose (LIT) medium*.*

### *In vitro *antileishmanial evaluation

#### Anti-promastigotes assay

The bioassay was performed using the AlamarBlue® method as previously described [23]. Promastigotes of *L. amazonensis* were grown at 26 °C in RPMI 1640 modified medium (Gibco BRL Life Technologies Inc., Gaithersburg, MD, USA) and supplemented with 10% heat-inactivated fetal bovine serum (FBS). Cultures in the logarithmic phase were seeded in sterilized 96-well microtiter plates (Corning™, Corning, NY, USA) (10^6^ parasites/ml) containing the samples dissolved in 1% DMSO at the suitable concentration to be tested in serial dilutions, and *Leishmania* medium (RPMI 1640) was used to reach a final volume of 200 μl per well; then 10% of AlamarBlue® was added to the entire plate. After an incubation of 72 h, the plate was checked visually using an inverted microscope, then analyzed using an Enspire multimode plate reader (PerkinElmer, Inc., Waltham, MA, USA) at excitation and emission wavelengths of 570 and 585 nm, respectively. Dose–response curves were plotted, and the concentration at which one half of the maximal inhibitory effect was obtained (IC_50_) was determined. The analyses were performed in triplicate.

#### Anti-amastigotes assay

The three molecules **1**-**3** were tested against the intra-macrophagic stage of *L. amazonensis* as previously described [15]. Briefly, in a 96-well, flat-bottom plate, macrophages of the J774A.1 cell line (American Type Culture Collection #TIB-67) were cultured at a density of 2 × 10^5^ cells/ml in RPMI 1640 medium supplemented with 10% heat-inactivated FBS and incubated for 1 h at 37 °C in a 5% CO_2_ atmosphere to allow almost complete attachment of the cells. The macrophages were then infected with *L. amazonensis* promastigotes in the stationary phase (7-day-old culture) at a ratio of 1:10 (macrophage:parasite) at a concentration of 2 × 10^6^ cells/ml and incubated at 37 °C in 5% CO_2_ for 24 h. Wells were washed with medium to remove external promastigotes. Thereafter, the infected macrophages were treated with the serially diluted concentrations of the pure compounds for 24 h, following which the medium was removed carefully and replaced by 30 μl of new medium containing 0.05% sodium dodecyl sulfate and the plate shaken for 30 s. Subsequently, 170 μl of Schneider’s medium was added to each well to give a final volume of 200 μl, and 20 μl of AlamarBlue® was added to the plate; the plate was then incubated at 26 °C for 72 h, following which the plates were analyzed using the same protocol as for the promastigotes assay.

#### *In vitro* evaluation on *T. cruzi* epimastigotes

The three molecules **1**-**3** were tested against epimastigotes of *T. cruzi.* Briefly, the molecules were dissolved in DMSO in 96-well plates and a serially diluted concentration was made in 100 μl of LIT medium supplemented with 10% heat-inactivated FBS. In all tests, a maximum of 1% DMSO was used to dissolve the highest dose of the compounds without inducing any effects on the parasites; epimastigotes in the logarithmic growth phase were then counted, adjusted to 5 × 10^5^ cells/ml, distributed on the previous 96-well plate and incubated at 27 °C for 72 h. The plate was observed under an inverted microscope after 72 h of incubation and analyzed statistically as described for the leishmanicidal test.

#### Assessment of cytotoxicity on macrophages

Cytotoxicity of the tested molecules was evaluated after 24 h using macrophages of the murine cell line J774.A1 (American Type Culture Collection #TIB-67). The cells were maintained in DMEM medium at 37 ˚C in a 5% CO_2_ humidified incubator. The viability of the macrophages was determined with the AlamarBlue assay and the emitted fluorescence was assayed in an Enspire microplate reader (PerkinElmer Inc.) at 570/585 nm. Briefly, 50 µl of macrophages was placed in a 96-well flat bottom plate at a density of 2 × 10^5^/ml in RPMI medium supplemented with 10% FBS without phenol. Cells were allowed to adhere for 15 min, and their progress was checked using a Leika inverted microscope (Leika Camera AG, Wetzlar, Germany). At the same time, a serially dilution of the test compounds was made in a deep 96-well plate with the same medium, and then 50 µl of each serially diluted molecule was added to each well of the 96-well flat bottom plate. Finally, 10% of AlamarBlue was added to each well. The plates were incubated at 37 °C in 5% CO_2_ for 24 h. Dose–response curves were plotted and the 50% cytotoxic concentrations (CC_50_) were obtained. The analyses were performed in triplicate.

### DNA condensation assay

Hoechst dye is a blue-fluorescent dye that stains condensed chromatin of apoptotic cells. To detect DNA condensation, the chromatin condensation/dead cell apoptosis kit was used (Invitrogen, Thermo Fisher Scientific, Waltham, MA, USA). Cells were incubated in the presence of the compounds to be tested at IC_90_. The IC_90_ values were determined using nonlinear regression of the AlamarBlue® curve. After 24 h of incubation, cells were harvested and resuspended in medium, following which 1 μl of Hoechst 33342 stock solution was added to parasites and incubated for 20 min. Results were analyzed by cell imaging using the Evos FL cell imaging system (Thermo Fisher Scientific). The experiments were repeated using a double-stain apoptosis detection kit (Hoechst 33342/PI; Thermo Fisher Scientific). This kit allows the identification of three groups in a cellular population: (i) dead cells, which exhibit high-red and low-blue fluorescence (as propidium iodide stain enters the nucleus); (ii) apoptotic cells, which will show high-blue fluorescence (as chromatin condenses); and (iii) healthy cells with only a low level of fluorescence. A positive control was also tested: miltefosine (*L. amazonensis)* and benznidazole (*T. cruzi).*

### Analysis of mitochondrial membrane potential

Promastigotes of *L. amazonensis* (2 × 10^6^ cells/ml) and epimastigotes (5 × 10^5^cells/ml) of *T. cruzi* were incubated for 24 h in the presence of the compounds to be tested, at IC_90_ and 26 °C, following which the cells were centrifuged, resuspended and transferred to black bottom 96-well plate. A 10-μl aliquot of JC-1 reagent (JC-1 Mitochondrial Membrane Potential Assay Kit; Cayman Chemical, Ann Arbor, MI, USA) was added to each well and the plates were incubated for 30 min. The switch of fluorescence from red to green emitted by both forms of the dye (monomer and aggregate) was measured using the Enspire microplate reader (PerkinElmer) at excitation/emission wavelengths of 560/595 and 485/535 for red and green, respectively; the decrease of the ratio was calculated as the percentage relative to the negative control. Cells images were captured using the Evos cell imaging system (Thermo Fisher Scientific), which is a fully integrated digital inverted microscope, at a magnification of ×40 and ×100. Untreated cells are used as the negative control, and miltefosine and benznidazole were used as the positive control.

### Analysis of adenosine triphosphate levels

The CellTiter-Glo® luminescent cell viability assay (Promega, WI, USA) was used to quantify the adenosine triphosphate (ATP) concentration. Cells of the kinetoplastid strains (for both *L. amazonensis* [2 × 10^6^ cells/ml] and *T. cruzi* [5 × 10^5^cells/ml]) were incubated for 24 h with the compounds to be tested, at IC_90_ and 26 °C. After incubation, the cells were centrifuged and resuspended in 100 μl. To this volume, we added 100 μl of the reactive in white 96-well plates. After incubation, the luminescence signal was measured using the Enspire multimode plate reader. The decrease in ATP level was expressed as a percentage relative to the negative control. Miltefosine and benznidazole were tested as the positive control for *L. amazonensis* and *T. cruzi*, respectively.

### Plasma membrane permeability assay

This assay was performed to evaluate the cell’s membrane permeability. Following the manufacturer’s recommendations, promastigotes of *L. amazonensis* and epimastigotes of *T. cruzi* were incubated in phosphate saline buffer in the presence of 1 μM of SYTOX® Green nucleic acid stain (Life Technologies, Waltham, MA, USA) and the IC_90_ of the different test compounds to be tested. Variation in fluorescence was measured every 15 min for 6 h using the Enspire multimode plate reader at excitation/emission wavelengths of 485/520. The percentage of relative permeabilization was calculated in reference to complete permeabilization realized by adding Triton X-100 (Sigma-Aldrich). In addition, both *L. amazonensis* and *T. cruzi* cells were incubated for 24 h with compounds **1**–**3**, miltefosine and benznidazole, and images were captured using the Evos cell imaging system.

### Oxidative stress

The reagent used for this assay was a fluorogenic probe to measure cellular oxidative stress upon oxidation by reactive oxygen species (ROS). To perform the test, cells were incubated with the compounds to be tested, at IC_90_ for 24 h. The cells were centrifuged and resuspended in a volume of 50 μl, following which CellROX™ Deep Red oxidative stress reagent (Thermo-Fisher Scientific) was added to 96-well plates at 5 mM. The plates were then incubated for 30 min at 37 °C. After incubation, the results were analyzed by cell imaging using the Evos cell imaging system. Cells treated with different compounds were compared with untreated control cells and the positive control. Fluorescence intensity was also measured using the Enspire multimode plate reader at excitation/emission wavelengths of 640/665 nm.

### Statistical analysis

All data are expressed as the mean ± standard deviation of at least three independent experiments. A statistical comparison was conducted using one-way analysis of variance. All analyses and the graphics were done using GraphPad Prism version 8.0 (GraphPad Software Inc., San Diego, CA, USA). Statistical significance was set at *P* < 0.05.

## Results

### *In vitro* activity and selectivity index

The cytotoxicity of compounds **1**–**3** was evaluated* in vitro* against the murine macrophage cell line and the CC_50_ was calculated and expressed in millimoles (µM). Leishmanicidal assays were also performed to determinate the IC_50_ of the three diterpenes under study against both the amastigote and promastigote stages of *L. amazonensis.* Values of IC_50_ against amastigotes and CC_50_ against murine macrophages allow the determination of the Selectivity Index (SI) on compounds **1**–**3**. The results are summarized in Table 1. Regarding cytotoxicity, CC_50_ values were found to range from 36.96 µM for compound **1** to 69.98 µM for compound **3**. Compared to miltefosine and benznidazole, the three isolated diterpenes exhibited a higher toxicity.

**Table 1:** Summary of in vitro leishmanicidal, trypanocidal and cytotoxic activity of compounds **1**–**3** isolated from the crude extract of *Dictyota spiralis*, and the Selectivity Index, compared to the positive controls miltefosine and benznidazole

Compound	CC_50_: murine macrophages (μM)	*Leishmania amazonensis*			*Trypanosoma cruzi*
		IC_50_: promastigote (µM)	IC_50_: amastigote (μM)	Selectivity index	IC_50_: epimastigote (μM)
**1**	36.96 ± 2.74a	18.89 ± 5.28b	0.97 ± 0.13a	37.76	13.11 ± 3.62b
**2**	52.05 ± 6.63b	18.95 ± 0.42b	2.03 ± 0.22b	25.56	18.32 ± 0.91c
**3**	69.98 ± 0.13c	36.81 ± 5.19c	3.88 ± 0.17c	18.02	35.28 ± 4.09d
**Miltefosine** (positive control for *Leishmania amazonensis*)	72.18c	6.47 ± 0.58a	3.11c	23.16	–
**Benznidazole** (positive control for *Trypanosoma cruzi* )	400d	–	–	–	6.95a

Values in table are presented as the mean ± standard deviation (SD). Means within compounds (rows) with different lowercase letters (a–d) are significantly different (*P* < 0.05) for each test

IC_50_, Inhibitory concentration that inhibits 50% of the growth of the tested parasite; CC_50_, cytotoxic concentration that reduces 50% of the murine macrophages’ viability; Selectivity Index (SI), CC_50_/IC_50_)

The in vitro activity of compounds **1**–**3** was evaluated against both the amastigote and promastigote stages of *L. amazonensis*. The results showed that their activity against the promastigote stage was lower than that of the reference drug. The IC_50_ value obtained for miltefosine was 6.47 µM and that of the compounds isolated from the *D. spiralis’* extract exhibited IC_50_ values ranging from 18.89 to 36.81 µM. The amastigote stage of *L. amazonensis* showed a greater sensitivity to compounds **1**–**3**, as presented in Table 1. Compounds **1** and **2** had a significantly stronger activity than miltefosine, with IC_50_ values of 0.97 and 2.03 µM for compounds **1** and **2**, respectively, and 3.11 µM for the reference drug. The SI values, calculated as the ratio between cytotoxicity and activity against the intracellular form of *L. amazonensis*, suggest that compounds **1** and **2**, which showed a higher selectivity to the parasite than miltefosine (reference drug), are of potential interest.

In vitro evaluation of trypanocidal activity exhibited IC_50_ values ranging from 13.11 to 35.28 µM for compounds **1**–**3**; in comparison benznidazole is active at an IC_50_ of 6.95 µM.

Table 2 summarizes the IC_90_ values obtained for each compound and for miltefosine and benznidazole. These concentrations were then used in the cell death mechanism assays.

**Table 2:** Summary of IC_90_ values obtained in vitro

Compound	*Leishmania amazonensis*: promastigote (μM)	*Trypanosoma cruzi*: epimastigote (μg/M)
**1**	39.47	75.43
**2**	34.94	104.08
**3**	74.87	139.90
**Miltefosine**	9.63	–
**Benznidazole**	–	25.59

### DNA condensation

**Fig. 2:**
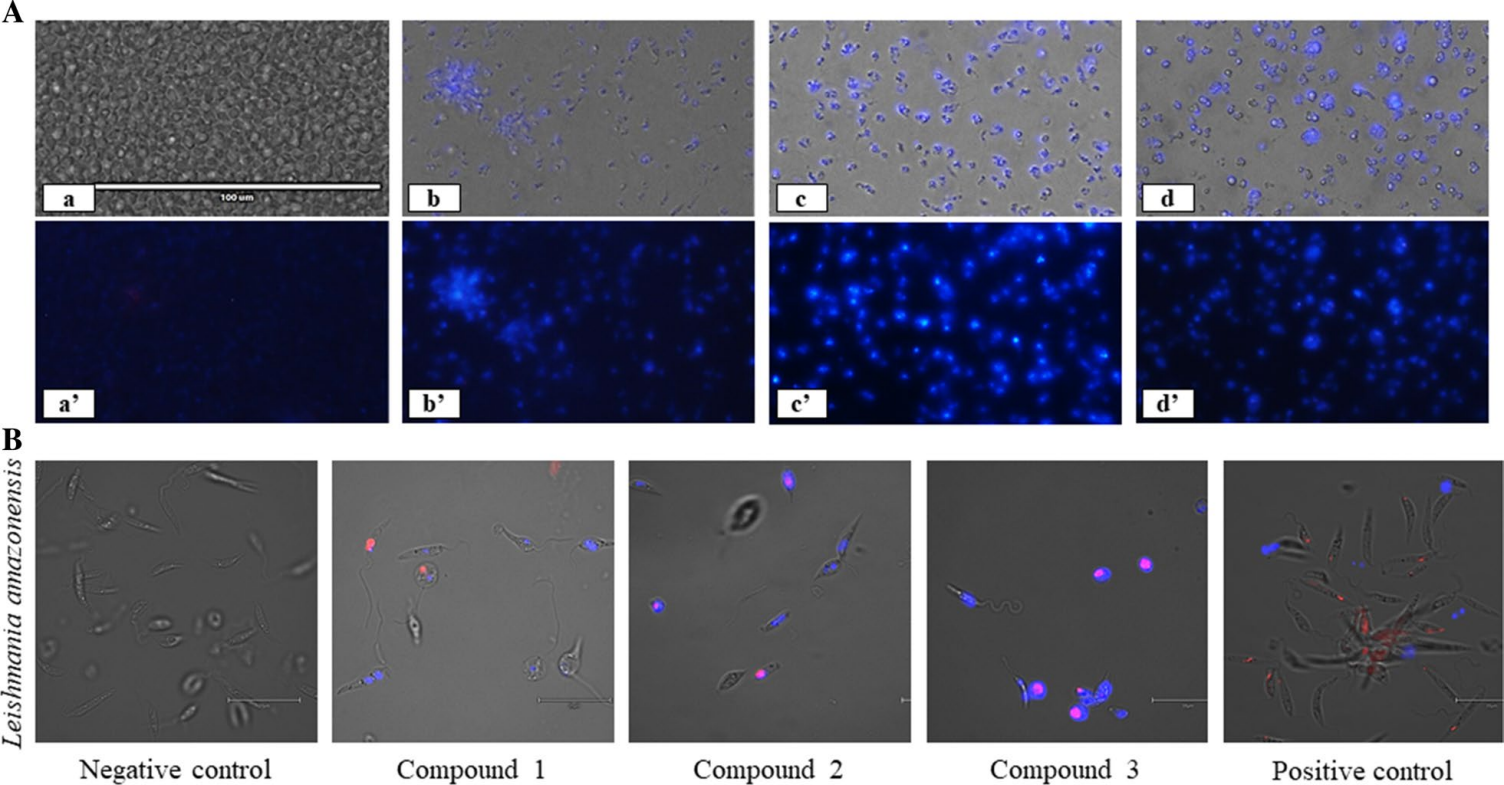
**A** Hoechst stain assay against promastigote stage *Leishmania amazonensis* incubated with IC_90_ of compounds **1**–**3** for 24 h. Images (×40) obtained using the EVOS FL Cell Imaging System (Thermo Fisher Scientific). Overlay is represented with (a–d) and without (a′–d′) visible channel. Shown are negative control cells with DMSO (a and a′), compound **1** (b and b′), compound **2** (c and c′) and compound **3** (d and d′). **B** Double-stain assay against promastigote stage *Leishmania amazonensis* incubated with IC_90_ of compounds **1**–**3** for 24 h. Miltefosine is used as the positive control and DMSO as the negative control. Images (×100) were obtained using the EVOS FL Cell Imaging System with overlay of visible, blue and red channel. The presence of Hoechst stain corresponds to the presence of chromatin condensation (blue) in treated cells. Red fluorescence corresponds to the staining by propidium iodide (PI), indicating dead cells

**Fig. 3:**
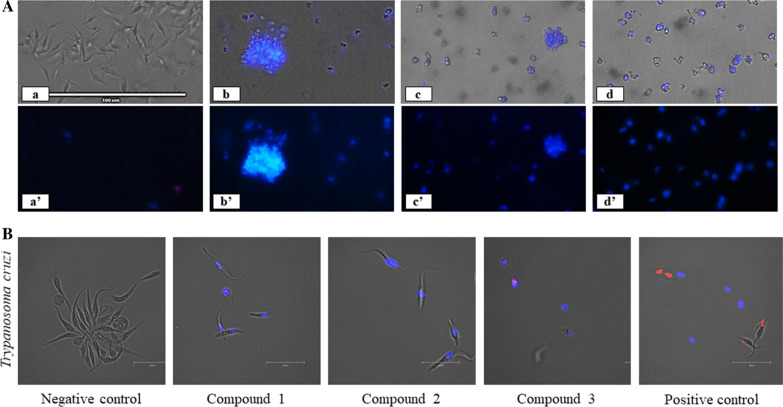
**A** Hoechst stain assay against epimastigote stage *Trypanosoma cruzi* incubated with IC_90_ of compounds **1**–**3** for 24 h. Images (×40) obtained from the EVOS FL Cell Imaging System. Overlay is represented with (a to d) and without (a′ to d) visible channel. Shown are negative control cells with DMSO (a and a′), compound **1** (b and b′), compound **2** (c and c′) and compound **3** (d and d′). **B** Double-stain assay against epimastigote stage *Trypanosoma cruzi* incubated with IC_90_ of compound **1**–**3** for 24 h. Miltefosine is used as the positive control and DMSO as the negative control. Images (×100) were obtained using the EVOS FL Cell Imaging System with overlay of visible, blue and red channel. The presence of Hoechst stain corresponds to the presence of chromatin condensation (blue) in treated cells. Red fluorescence corresponds to staining by PI, indicating dead cells

DNA condensation is an important cellular event, providing evidence of apoptotic cell death. Hoechst dye is a blue-fluorescent dye that stains condensed chromatin. In this assay, a normally functioning live cell exhibits a light-blue color and early apoptotic cells show a bright-blue fluorescence that indicates the presence of condensed chromatin. Also, using propidium iodine (PI), it is possible to distinguish dead cells, which stain red when PI penetrates the nucleus. The results of staining were evaluated using cell imaging. Different microscopy images are regrouped in Fig. 2 (for *L. amazonensis*) and Fig. 3 (for *T. cruzi*). As observed in Fig. 2A, compared to the negative control, all compounds induced an increase in blue fluorescence after incubation with promastigotes of *L. amazonensis* cells. A clear reduction in cell number is also evident. Similar results were obtained with *T. cruzi* and the Hoechst dye, as shown in Fig. 3A.

A magnification of ×100 associated to the use of PI enabled the images shown in Fig. 2B of double-stained *L. amazonensis* cells incubated with the negative control, compounds **1**–**3** and the positive control (miltefosine). Cells exhibiting no fluorescence (negative control) are healthy cells; those showing a superposition of blue and red fluorescence, such as cells treated with compounds **2** and **3**, suggest a late apoptotic stage; and cells with only red fluorescence are dead cells, somewhat like the image taken after exposure to miltefosine. The same results are observed in Fig. 3B where epimastigote stage *T. cruzi* are incubated with compounds **1**–**3** and benznidazole is the positive control.

As shown in Figs. 2 and 3, the results for both parasites are similar, suggesting that the tested compounds behave in the same way toward cells of these two kinetoplastids.

### Alteration of the mitochondrial membrane potential

**Fig. 4:**
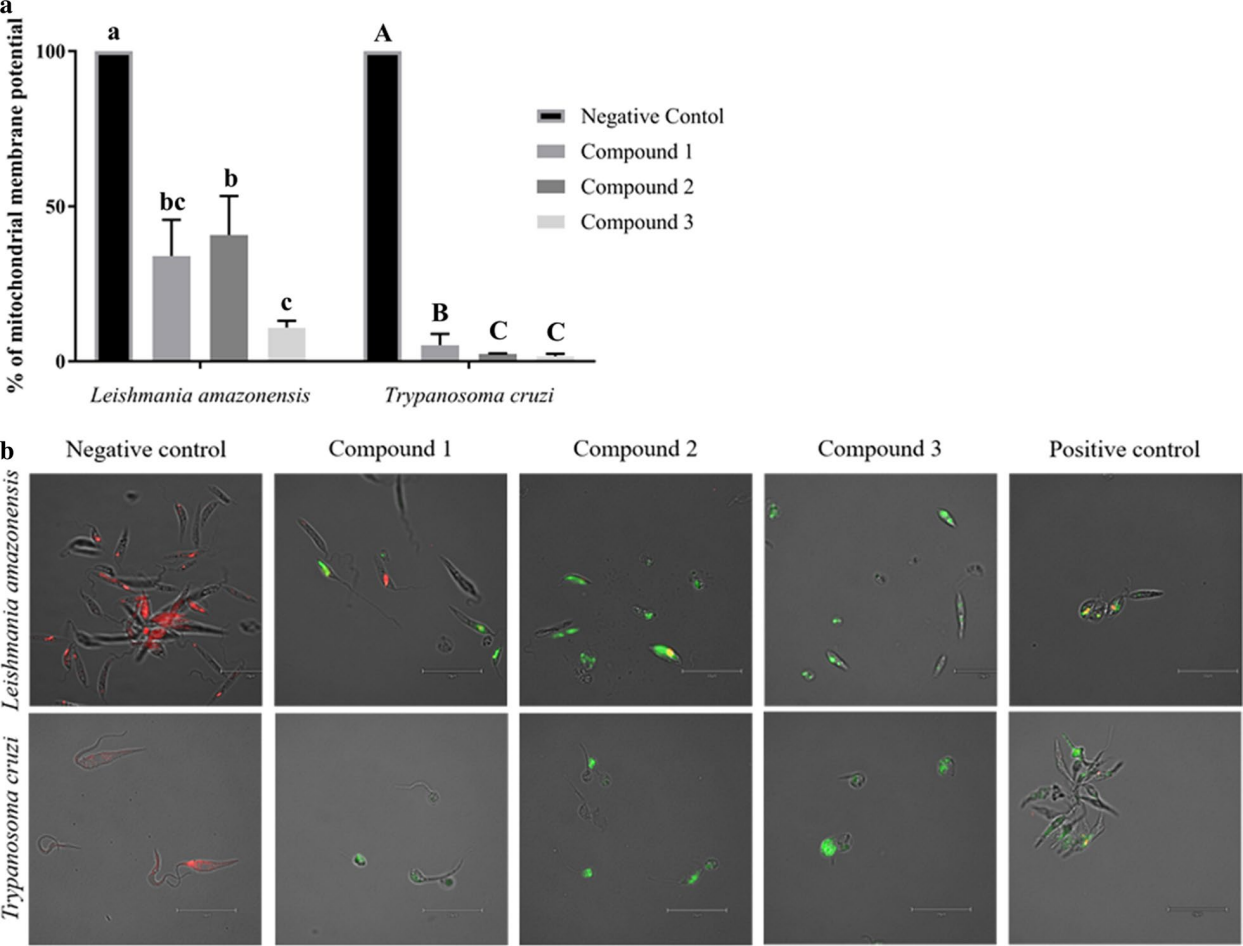
**a** Bar graph showing changes in the mitochondrial membrane potential (ΔΨm) of *L. amazonensis* and *T. cruzi* after 24 h of incubation with the IC_90_ of compounds **1**–**3** compared to the negative control (DMSO). Differences between the values were assessed using one-way analysis of variance. Data are presented as the mean ± standard deviation (SD). Lowercase letters a–c or uppercase letters A–C indicate that means within compounds with different letters are significantly different (*P* < 0.05). **b** Images (×100) are representative of the parasites observed in the performed experiments using the EVOS FL Cell Imaging System

Disruption of mitochondrial functions induced by the incubation of *L. amazonensis* and *T. cruzi* with compounds **1**–**3** was evaluated using the mitochondrial membrane potential as a measure. JC-1 dye was used in this assay. In the case of normal mitochondrial membrane potential, the reactive dye will form aggregates that emit red fluorescence; a collapsed potential induces the monomeric form with green fluorescence. This parameter is expressed as the absolute red/green ratio of fluorescence. We considered the values of the negative controls to be the normal values, and therefore the collapse of mitochondrial membrane potential is expressed as a percentage (see Fig. 4a). This parameter is involved in energy storage and is an important indicator of cell health and viability [16].

The results demonstrate that exposing parasite cells to all tested compounds (**1**–**3**) induced an important decrease in mitochondrial membrane potential (ΔΨm) of both *L. amazonensis* and *T. cruzi*. These three diterpenes isolated from the brown seaweed *D. spiralis* strongly affected *T. cruzi*, with a decrease in membrane potential of > 95%. The effect seems to be more moderated in *L. amazonensis*. Compound **3** had the strongest effect on *L. amazonensis*, effecting a decrease in mitochondrial potential of > 80% compared to compounds **1** and **2** that induced a decrease in mitochondrial potential of 34.0 and 40.7%, respectively.

The measured results are also supported by cell imaging with miltefosine added as a positive control for *L. amazonensis* and benznidazole as a positive control for *T. cruzi.* Microscopy images overlaying green, red and transmitted light at a magnification of ×100 of parasites incubated with positive control, negative control and compounds **1**–**3** are shown in Fig. 4B. The control was characterized by the domination of red fluorescence, thereby corroborating a high mitochondrial potential and healthy cells. In comparison, cells treated with compounds **1**–**3** exhibited a predominance of green fluorescence in the case of *T. cruzi* and a mix between green and red (orange color) for *L. amazonensis* due to a lower effect on the latter, especially for compound **2**.

### ATP concentration

**Fig. 5:**
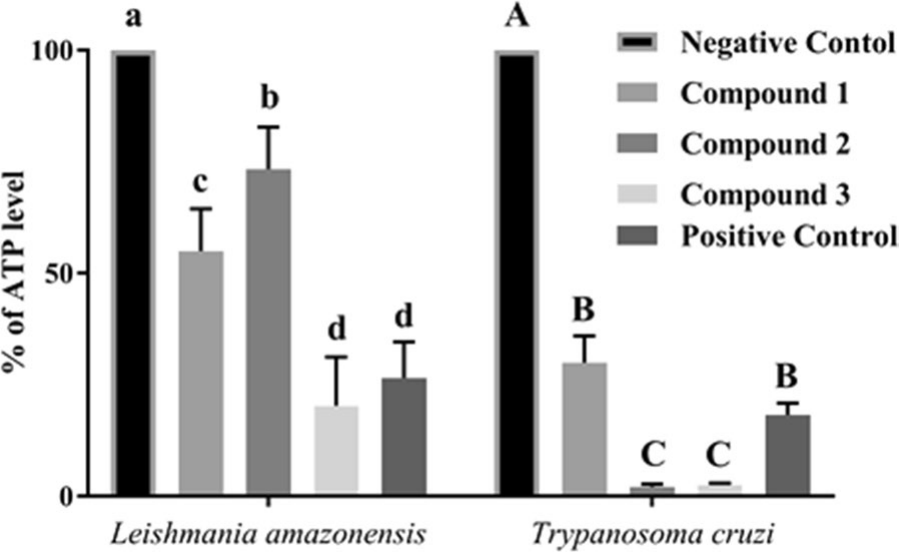
Effect of compounds **1**–**3**, miltefosine and benznidazole on ATP level of *L. amazonensis* and *T. cruzi* using the CellTiter-Glo® luminescent cell viability assay. The results are given in percentage relative to the negative control (DMSO). Cells were treated by the IC_90_ concentration for 24 h. Values are given as mean ± SD. Lowercase letters a–c or uppercase letters A–C indicate that means within compounds with different letters are significantly different (*P* < 0.05)

Quantification of ATP level in control cells (for both *T. cruzi* and *L. amazonensis*) and in cells treated for 24 h with IC_90_ of the different compounds isolated from *D. spiralis* are shown in Fig. 5. Miltefosine was used as the positive control for *L. amazonensis* and benznidazole as the positive control for *T. cruzi.* As can be observed, untreated cells (negative control) represent 100% of the ATP level. The effect induced by all compounds and the positive control was a decrease in ATP concentration for both parasites. *Trypanosoma cruzi* cells incubated with compounds **2** and **3** lost > 95% of their ATP concentration. The effects of compound **1** and benznidazole were more moderate, with a decrease in ATP concentration of > 70 and > 80%, respectively. For *L. amazonensis*, the effect was less important, especially for compound **2**, with a loss of 26.6%. Regarding compound **3**, the results obtained are similar to those obtained with miltefosine, with no significant difference between them.

### Membrane permeability alteration

**Fig. 6:**
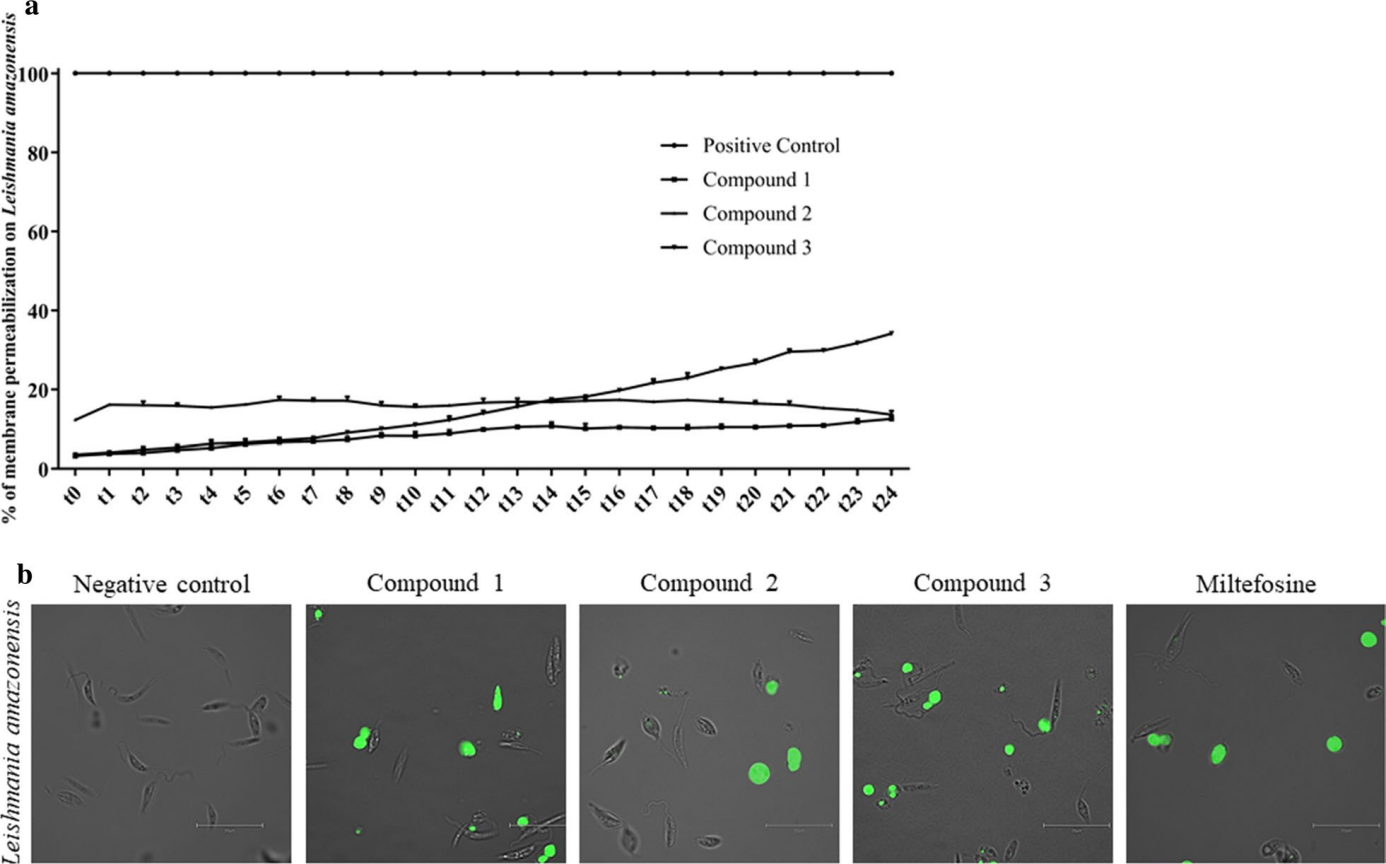
**a** Effect of compounds **1**–**3** on *L. amazonensis’* plasma membrane permeability. Results are expressed as a percentage of membrane permeabilization compared to the total permeabilization obtained with Triton X-100. Values are given as the mean of three repetitions with SD bars at each measurement point. **b** Images (×100) are representative of *L. amazonensis* cells observed in the performed experiments with 24 h incubation using the EVOS FL Cell Imaging System. DMSO was used as the negative control contains DMSO and miltefosine was used as the positive control. Fluorescence was measured at 15-min intervals for 6 h (*t24*)

One of the detectable cellular events of a necrotic cell death is the rupture of the plasma membrane. This event can be detected by evaluating plasma membrane permeabilization. We performed the SYTOX Green dye assay to detect effect of compounds **1**–**3** on membrane permeability. This green-fluorescence dye has a high affinity for DNA. Triton X-100 was used as a positive control in light of its known effect to cause 100% membrane permeabilization. Values of fluorescence were measured each 15 min for 6 h. The results of this assay are summarized in Fig. 6 for *L. amazonensis .*

As can be observed, all of the tested molecules did not alter the membrane permeability for at least the first 6 h of incubation. Compound **3** exhibited the highest percentage of membrane permeabilization compared to the positive control, with increasing values reaching 35% after 6 h of incubation. For compounds **1** and **2**, a slight fluctuation was noted, but always at values of < 20% compared to Triton X-100 (100% permeabilization).

The measured values were supported by microscopy images, with miltefosine considered to be the reference drug and with a 24-h incubation. As can be observed in images regrouped Fig. 6b, the effect induced by the three diterpenes was very similar to that of miltefosine. No morphological changes of parasite can be distinguished, and cell shape is regular compared to negative untreated control.

**Fig. 7:**
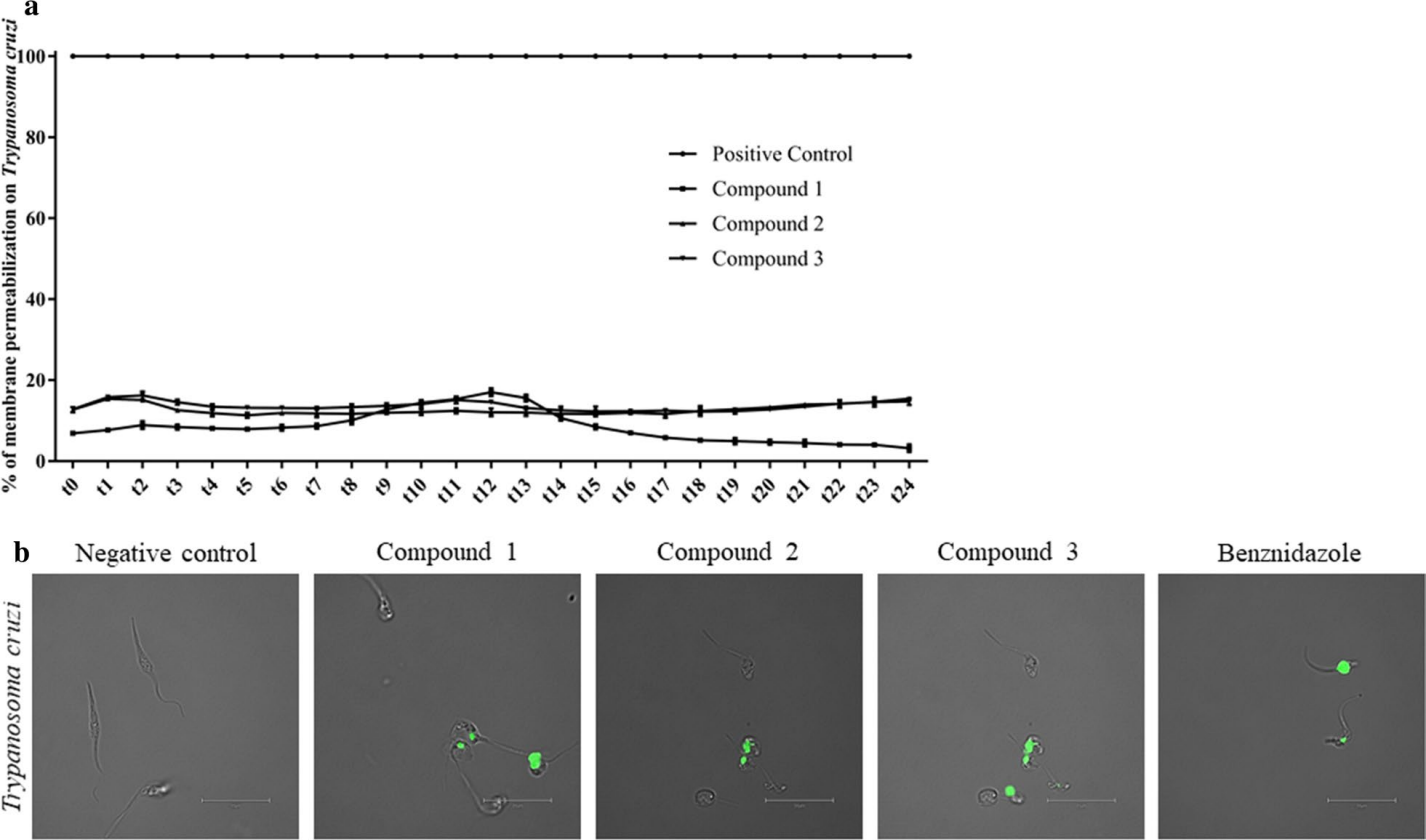
**a** Effect of compounds **1**–**3** on *T. cruzi* plasma membrane permeability. Results are expressed as a percentage of membrane permeabilization compared to the total permeabilization obtained with Triton X-100. Values are given as the mean of three repetitions with SD bars at each measurement point. **b** Images (×100) are representative of *T. cruzi* cells observed in the performed experiments with 24 h incubation using an EVOS FL Cell Imaging System Negative control contains DMSO, Benznidazole is used as positive controls

Variations in membrane permeabilization induced by compounds **1**–**3** in *T. cruzi* cells are shown in Fig. 7a. According to the results, compound **1** induced a slight increase of fluorescence after 2 h of incubation but this increase was followed with a decrease at the end of incubation. For compounds **2** and **3**, the evolution though incubation remained almost linear, and for all compounds, the values of plasma membrane permeabilization were < 20%

Images captured by the EVOS FL Cell Imaging System at ×100 magnification, 24-h incubation are grouped Fig. 7b. Compared to benznidazole (positive control for *T. cruzi*) a similar effect was induced by compounds **1**–**3** on the plasma membrane. Morphological changes were more remarkable, with irregular parasite shape, rounded cells and no flagella in some cases.

### Oxidative stress

Accumulation ROS at the cellular level is a good indicator of oxidative stress and is precursor to a chain reaction leading damage at the cellular level to proteins, nucleic acids, lipids, membrane and organelles. These cellular alterations can lead to activation of the cell death process. As such, ROS play an important role in cell signaling and the apoptosis pathway [17]. An intracellular excess of ROS can be detected by the cell-permeable CellROX™ Deep Red reagent, which is a fluorogenic ROS sensor. In its reduced state, the CellROX™ Deep Red reagent is nonfluorescent; however, upon oxidation by ROS, it exhibits bright near-infrared fluorescence that serves as a direct measurement of the ROS levels in live cells.

**Fig. 8:**
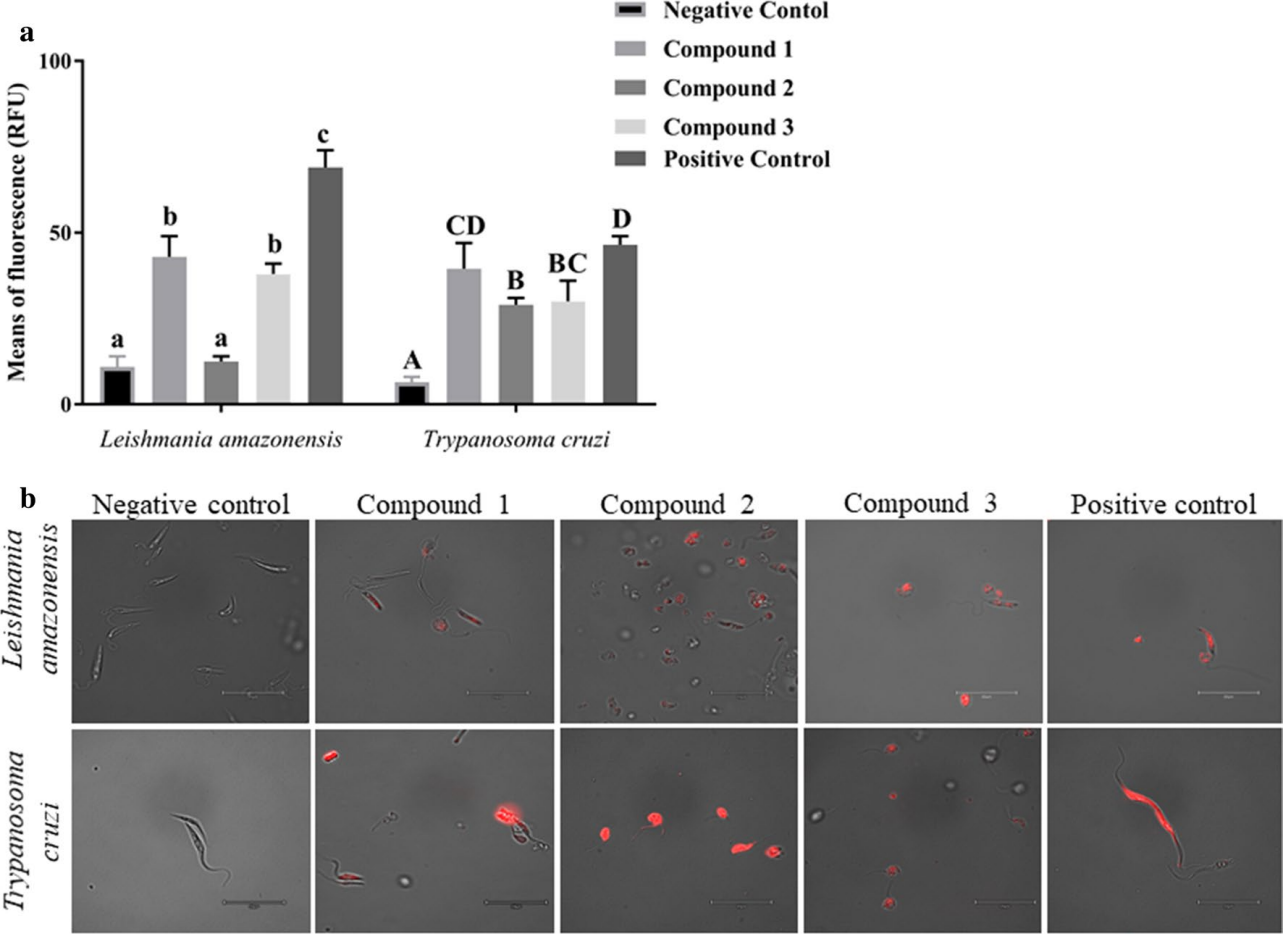
**a** Reactive oxygen species accumulation assay. Bar graph represents means of fluorescence emission in relative fluorescence units (*RFU*) of *L. amazonensis* and *T. cruzi* after 24 h of incubation with the IC_90_ of compounds **1**–**3** compared to the negative control (DMSO). Miltefosine and benznidazole were used as positive controls. Differences between the values were assessed using one-way analysis of variance. Data are presented as the mean ± SD. owercase letters a–c or uppercase letters A–C indicate that means within compounds with different letters are significantly different (*P* < 0.05). **b** Images (×100) are representative of the parasites observed in the performed experiments using the EVOS FL Cell Imaging System

To evaluate any variation in ROS accumulation, we measured fluorescence using the Enspire multimode plate reader. The results are shown Fig. 8a. The incubation of *L. amazonensis* cells with compound **2** induced a slight increase in fluorescence but the increase was not significant compared the negative control containing DMSO. The effect was more remarkable for compounds **1** and **3** and miltefosine. For *T. cruzi* cells, there was a significant increase in fluorescence for all compounds tested, as well as for benznidazole, compared to the negative control.

The measured values are supported by microscopy images using the EVOS FL Cell Imaging System at a magnification of ×100. Images are regrouped in Fig. 8b. As can be seen in the microscopy images, an accumulation of red fluorescence is present in all treated parasites due to the presence of ROS. This is indicator of oxidative stress induced by compounds **1**–**3**. A morphological modification of the parasite, which is associated to the effect of compounds on the cytoskeleton of both *L. amazonensis* and *T. cruzi*, was also observed.

## Discussion

From a chemical point of view, the three compounds isolated from the crude extract of *D. spiralis* can be divided into two main groups: compounds **1** and **2** with a dictyane skeleton, and compound **3** with a dolabellane skeleton. All three metabolites belong to the diterpene group.

Brown seaweeds (phaeophyta) belonging to order Dictyotales have emerged as an exceptionally rich source of terpenoid compounds, with more than 300 diterpenes from at least 35 species worldwide having been identified in the family Dictyotaceae [18].

Dolabellane-like compounds have been isolated from several marine organisms and have been associated to different biological activities, such as antiplasmodic [19] and antibacterial [20] activities and, as also shown in the present study, to leishmanicidal and trypanocidal capacities [10]. Other biological activities have also been associated to compounds with a chemical structure based on the dictyane skeleton, such as the antifouling activity exhibited by dictyol E, dictyol C and pachydictyol A when tested against marine invertebrate larvae [21] and antimicrobial [12] and cytotoxic effects [22], as well as antikinetoplastid capacity, as proven in the present study.

Pachydictyol C (compound **1**) was first isolated from the brown alga *D. dichotoma* collected along the coast of the Red Sea in Egypt. It has been shown to have* in vitro* cytotoxicity against breast carcinoma tumor cell line without showing any antitumoral or antimicrobial capacity [12].

Dictyol E (compound **2**) is a secondary metabolite found in *Dictyota* species and is used to defend the cell from herbivores [14]. This diterpenoid was first identified in 1987 from the brown seaweed *D. dichotoma*, and its capacity as herbivore repulsive was proven against fish and urchins [23]. Dictyol E has been also associated to some biological activities, such as moderate cytotoxic activity against some cancer cell lines [22] and as inhibitor of diacyl glycerol transferase activity, a factor involved in triglyceride accumulation in organisms [24].

Compound **3**, 3,4-epoxy-7,18-dolabelladiene, has been identified in *D. dichotoma* collected along the Italian coast and later in *Dilophus spiralis* (synonym *D. spiralis*) [20, 25, 26].

In the present study, the leishmanicidal and trypanocidal activities of compounds **1**–**3** were proven using the Alamarblue method, which allowed us to determinate both the IC_50_ and IC_90_ of these molecules. Pachydictyol C, Dictyol E and 3,4-epoxy-7,18-dolabelladiene belong to the large group of terpenes showing antikinetoplastid capacity. Although this study is the first to report the leishmanicidal and trypanocidal capacity of these molecules, other structures have been associated to these capacities. The majority of examples are associated to terpenes isolated from aromatic and medicinal plants. For example nerolidol, a sesquiterpene present in essential oils, is leishmanicidal against *Leishmania* spp. [27], and oleanolic and maslinic acids, both triterpenes extracted from the olive leaf, has been found to show interesting activity against *Leishmania* spp. Some terpenes extracted from seaweeds have also been tested for their antikinetoplastid capacity.

Dos Santos et al. [28] studied 4-acetoxydolastane diterpene from the Brazilian brown alga *Canistrocarpus cervicornis* as an antileishmanial agent. Dolabelladienetriol, which is structurally similar to compound **3**, was isolated from the brown seaweed *D. pfaffii* and showed a capacity to inhibit infection by *L. amazonensis* [29] with an IC_50_ of 44 µM. These results highlight our interest in compounds isolated from the crude extract of *D. spiralis*: the IC_50_ of compounds **1** and **2** was shown to be twofold lower than that of dolabelladienetriol. Investigations have also been performed on the trypanocidal effect of compounds isolated from seaweeds. Eleganolone, a diterpene from the alga *Bifurcaria bifurcate*, is a compound isolated from seaweeds that has been associated to trypanocidal activity [30]. This molecule was active against *T. cruzi* with an IC_50_ of 58 µM.

All of the examples associated above are associated to properties such as cytotoxicity against murine macrophages, and the SI of compounds **1**–**3** suggests the need to elucidate their mechanism of action on parasite cells. To this end, we performed several assays to detect different events indicative of the cell death pathway. Miltefosine was taken as the reference drug for *L. amazonensis* and benznidazole for *T. cruzi.* These molecules are already known to induce an apoptotic-like cell death in this parasite [31, 32].

An apoptotic-like cell death in trypanosomatids (which include *Leishmania* spp. and *Trypanosoma* spp.) has been described based on apoptotic phenotype. Various events can be detected, such as caspase activation, DNA fragmentation and chromatin condensation, blebs in the plasma membrane, cell shrinkage, mitochondrial membrane potential (ΔΨm) loss and phosphatidylserine exposure, ROS generation, among others.

In the present study, we first analyzed DNA condensation, an indicator of an apoptotic cell death, using the Hoechst stain assay, and the results obtained support the hypothesis of an apoptotic cell death. The behavior of the cells toward compounds **1**–**3** was very similar to that of miltefosine and benznidazole.

An apoptotic cell death in organisms belonging to kinetoplastids is associated with mitochondrial dysfunction and involves the collapse of the mitochondrial membrane potential. This event was detected in *L. amazonensis* and *T. cruzi* using the JC-1 cell line. The results confirmed the decrease induced by all compounds for both parasites. Our investigation of ATP level in association with the collapse of the mitochondrial potential showed that mitochondrial dysfunction can be assigned to the intrinsic pathway* via* mitochondria [33]. The integrity of plasma membrane permeability was also evaluated, and the results suggested a moderate alteration of plasma membrane permeability; a similar effect was seen with benznidazole and miltefosine. To complete our elucidation of the cell death mechanism induced by compounds **1**–**3**, in comparison to miltefosine and benznidazole, we detected the accumulation of ROS. ROS are involved in oxidative stress and their regulation is crucial for cell integrity. Our results highlight that compounds **1**–**3** as well as the positive controls induced the accumulation of ROS at the cellular level.

All of these events corroborate the hypothesis of an apoptotic-like cell death that is induced by the three compounds isolated from the crude extract of *D. spiralis*. As such, our results should encourage further assays and* in vivo* tests with the aim to evaluate the tolerance of living organisms to these molecules in the perspective of therapeutical application.

## Conclusions

The results obtained in this study detail the effect of three diterpenes isolated from *D. spiralis* on parasitic cells of *L. amazonensis* and *T. cruzi.* The experiments revealed structural, morphological and metabolic changes in the parasitic cells treated with these compounds. We were able to highlight the collapse of the mitochondrial potential, accumulation of ROS, chromatin condensation, maintenance of membrane permeability, decrease in ATP concentration and change in the cell’s morphology.

Based on these results from the different assays performed, we conclude that diterpenes 1–3, with dictyol and dolabellane skeletons, isolated from the brown algae *D. spiralis* induced, in the tested parasitic strains, events suggesting an apoptotic-like cell death. These preliminary results are promising and encourage further study of these compounds as therapeutic agents against both *L. amazonensis* and *T. cruzi.*

## Supplementary Information

**Additional file 1.**  Collection site, biological material, and isolation and purification procedure for metabolites from *Dictyota spiralis*. ^1^H and ^13^C NMR spectra of compounds 1-3.

## Data Availability

All data generated or analyzed during this study are included in this published article.

## Abbreviations

ATP: Adenosine triphosphate
CC: Cytotoxic concentration
DMSO: Dimethyl sulfoxide
IC: Inhibitory concentration
ROS: Reactive oxygen species
SI: Selectivity Index
