# Supplementary material for: Apoptosis-like cell death upon kinetoplastid induction by compounds isolated from the brown algae Dictyota spiralis
Source: Parasit Vectors. 2021 Apr 12;14:198. doi: 10.1186/s13071-021-04693-7 (PMC8042727; doi:10.1186/s13071-021-04693-7)
Supplement: Supplementary file 1 — Additional file 1. Collection site, biological material, and isolation and purification procedure for metabolites from Dictyota spiralis. 1H and 13C NMR spectra of compounds 1-3. [file 13071_2021_4693_MOESM1_ESM.docx]

**Additional Material**

Apoptosis-like cell death on kinetoplastid induces by compounds isolated from the brown algae *Dictyota spiralis*

**Olfa Chiboub^1,2,3,4^*, Ines Sifaoui^1,2,5^, Manef Abderrabba^4^, Mondher Mejri^4^, José Javier Fernández^3,6^, Ana R. Díaz-Marrero^3^, Jacob Lorenzo-Morales^1,2,5^, and José E. Piñero^1,2,5^**

^1^ Instituto Universitario de Enfermedades Tropicales y Salud Pública de Canarias, Universidad de La Laguna (ULL), Avda. Astrofísico Fco. Sánchez s/n, 38203 La Laguna, Tenerife, Spain

^2^ Departamento de Obstetricia y Ginecología, Pediatría, Medicina Preventiva y Salud Pública, Toxicología, Medicina Legal y Forense y Parasitología, Universidad de La Laguna (ULL), Tenerife, Spain

^3^ Instituto Universitario de Bio-Orgánica Antonio González, Universidad de La Laguna (ULL), Avda. Astrofísico Fco. Sánchez 2, 38206 La Laguna, Tenerife, Spain

^4^ Laboratoire Matériaux-Molécules et Applications, La Marsa, University of Carthage, Carthage, Tunisia

^5^ Red de Investigación Cooperativa en Enfermedades Tropicales (RICET)

^6^ Departamento de Química Orgánica, Universidad de La Laguna (ULL), Avda. Astrofísico Fco. Sánchez s/n, 38203 La Laguna, Tenerife, Spain

* Correspondence and requests for materials should be addressed to

Table of Contents

|  | Contents | Page |
| --- | --- | --- |
| 1 | **Collection site** | S2 |
| 2 | **Biological material** | S3 |
| 3 | **Isolation and purification procedure for metabolites from *Dictyota* *spiralis*** | S4 |
| 4 | **Figure S1.** ^1^H NMR spectrum of pachydictyol C (**1**) in CDCl_3_ (600 MHz, 300 K) | S5 |
| 5 | **Figure S2.** ^13^C NMR spectrum of pachydictyol C (**1**) in CDCl_3_ (150 MHz, 300 K) | S6 |
| 6 | **Figure S3.** ^1^H NMR spectrum of dictyol E (**2**) in CDCl_3_ (600 MHz, 300 K) | S7 |
| 7 | **Figure S4.** ^13^C NMR spectrum of dictyol E (**2**) in CDCl_3_ (150 MHz, 300 K) | S8 |
| 8 | **Figure S5.** ^1^H NMR spectrum of 3,4-epoxy-7,18-dolabelladiene (**3**) in CDCl_3_ (600 MHz, 300 K) | S9 |

**Collection site**

The collection site, located at the northern coast of Tunisia, is called Tabarka (36°57′16″N, 8°45′29″E). The area is characterized by a 10-km-long coastline of a specific biotope between sea and mountain with the presence of embayments, long extensions, headlands, pocket beaches, sand dunes and sea-cliffs. All these elements allow a wide biodiversity in fauna, flora and, also, marine life.


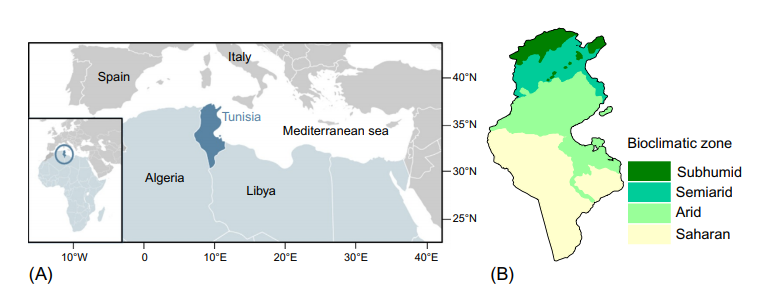


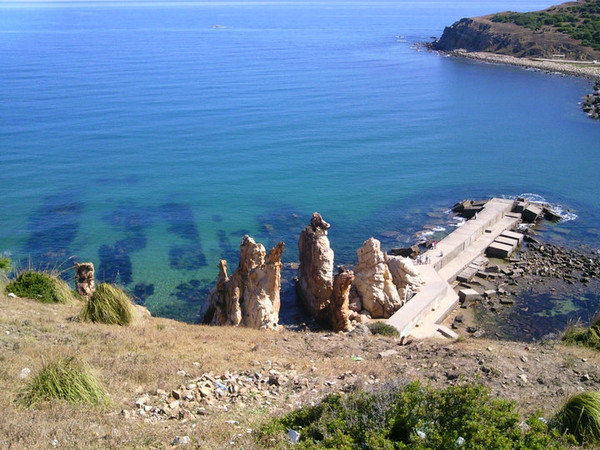


(C)

**Figure S1.** Map of Tunisia in the Mediterranean Basin (**A**), bioclimatic zones and location of Tabarka (**B**), and photograph of the collection site called “The Needles” (Tabarka, Tunisia) (**C**).

**Biological material**

Specimens of the brown alga *Dictyota spiralis* were collected by hand between December 2015 and March 2016 at 1.5 m of depth. Collection and identification were supervised by Dr. L. Ktari, from the National Institute for Marine Sciences and Technologies (INSTM). Voucher specimens were assigned the codes: OC-04042017-1, OC-13042017-1, OC-17042017-1.


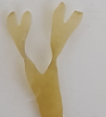

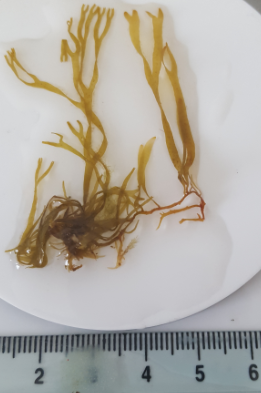


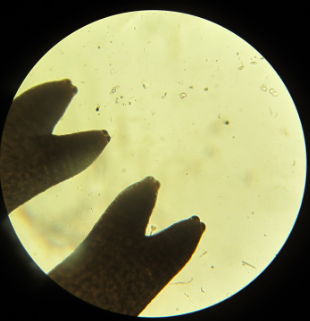

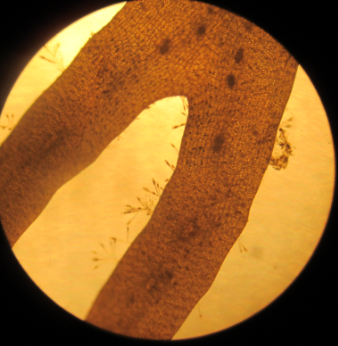


**Figure S2.** Images of *Dictyota spiralis* (Brown alga, Class Phaophyceae). The size of this species is inferior to 30 cm. The thallus is very ramified and formed of strips ribbons or thongs. Observed ramifications are dichotomic with absence of longitudinal median veins.

**Isolation and purification procedure for metabolites from *Dictyota* *spiralis***

The dried and powdered algal material (190 g) was extracted by maceration at room temperature in dichloromethane (DCM). The solvent was renewed several times for a maximized extraction. DCM solution was ﬁltered and evaporated with rotatory evaporator at 40 ºC to give 10 g of crude extract. 5 g of the obtained extract was fractionated as indicated in the following scheme:

**Figure S1.** ^1^H NMR spectrum of pachydictyol C (**1**) in CDCl_3_ (600 MHz, 300 K)

**Figure S2.** ^13^C NMR spectrum of pachydictyol C (**1**) in CDCl_3_ (150 MHz, 300 K)

**Figure S3.** ^1^H NMR spectrum of dictyol E (**2**) in CDCl_3_ (600 MHz, 300 K)

**Figure S4.** ^13^C NMR spectrum of dictyol E (**2**) in CDCl_3_ (150 MHz, 300 K)

**Figure S5.** ^1^H NMR spectrum of 3,4-epoxy-7,18-dolabelladiene (**3**) in CDCl_3_ (600 MHz, 300 K)
